# Formulation and Evaluation of Microwave-Modified Chitosan-Curcumin Nanoparticles—A Promising Nanomaterials Platform for Skin Tissue Regeneration Applications Following Burn Wounds

**DOI:** 10.3390/polym12112608

**Published:** 2020-11-06

**Authors:** Hafiz Muhammad Basit, Mohd Cairul Iqbal Mohd Amin, Shiow-Fern Ng, Haliza Katas, Shefaat Ullah Shah, Nauman Rahim Khan

**Affiliations:** 1 Department of Pharmaceutics, Faculty of Pharmacy, Gomal University, DIKhan 29050, KPK, Pakistan; basitkhan053@gmail.com (H.M.B.); shefaatbu@gmail.com (S.U.S.); 2 Gomal Centre for Skin/Regenerative Medicine and Drug Delivery Research (GCSRDDR), Faculty of Pharmacy, Gomal University, DIKhan 29050, KPK, Pakistan; 3 Centre for Drug Delivery Technology, Faculty of Pharmacy, Universiti Kebangsaan Malaysia, Kuala Lumpur 50300, Malaysia; mciamin@ukm.edu.my (M.C.I.M.A.); nsfern@ukm.edu.my (S.-F.N.); haliza.katas@ukm.edu.my (H.K.)

**Keywords:** modified chitosan, curcumin, microwave, nanoparticles

## Abstract

Improved physicochemical properties of chitosan-curcumin nanoparticulate carriers using microwave technology for skin burn wound application are reported. The microwave modified low molecular weight chitosan variant was used for nanoparticle formulation by ionic gelation method nanoparticles analyzed for their physicochemical properties. The antimicrobial activity against *Staphylococcus aureus* and *Pseudomonas aeruginosa* cultures, cytotoxicity and cell migration using human dermal fibroblasts—an adult cell line—were studied. The microwave modified chitosan variant had significantly reduced molecular weight, increased degree of deacetylation and decreased specific viscosity. The nanoparticles were nano-sized with high positive charge and good dispersibility with entrapment efficiency and drug content in between 99% and 100%, demonstrating almost no drug loss. Drug release was found to be sustained following Fickian the diffusion mechanism for drug release with higher cumulative drug release observed for formulation (F)2. The microwave treatment does not render a destructive effect on the chitosan molecule with the drug embedded in the core of nanoparticles. The optimized formulation precluded selected bacterial strain colonization, exerted no cytotoxic effect, and promoted cell migration within 24 h post application in comparison to blank and/or control application. Microwave modified low molecular weight chitosan-curcumin nanoparticles hold potential in delivery of curcumin into the skin to effectively treat skin manifestations.

## 1. Introduction

Being the largest human organ, skin is often easily susceptible to defects sometimes being congenital defect and mostly by burn, trauma or diseases which have long lasting effect on the wellbeing and psychology of the patient. According to WHO statistics, globally 180,000 people die annually due to skin burn injuries only [1]. To date, autografts, allografts and xenografts has been used for the purpose. Although they are able to promote wound healing but they have inherent demerits of limited availability, high expenses, immune incompatibility, risk of infection and shortage of donor sites [2,3,4]. These demerits gave birth to developing skin scaffolds able to hasten the skin regeneration process by engineering skin substitutes made of different biodegradable polymers and their composites with aided medicaments. An artificial extracellular matrix is thus developed impregnated with skin healing hastening agents [5,6]. The process of tissue engineering enables self-healing potential of human body for regeneration of lost, damaged or injured tissues/organs which is enabled by creating suitable cell environment for its survival and functional achievements [5,6]. Various attempts has been made to formulate/develop skin substitutes to hasten the normal wound healing process including hydrogels [7], sponges [8], polymeric nanoparticles [9], nanofibers [10] and polymeric films [11] etc.

Chitosan has been extensively studied and finds extensive applications in the field of biomedical engineering and drug delivery to construct tissue scaffolds [12,13], as an excipients in drug delivery carriers [14,15], in gene delivery [16], and wound healing [13] and can easily be processed into gels [17], membranes [18], nanofibers [19], beads [20], microparticles [21], nanoparticles [22], scaffolds [23] and sponges [24]. Chitosan promotes cell proliferation, activates macrophages, stimulates tissue reorganization, promotes fibroblast proliferation, promotes collagen deposition, enhances increased production of hyaluronic acid at wound site and also acts as hemostatic agent, thus helps in wound healing with minimal scar formation [25,26]. The physicochemical properties of chitosan i.e., molecular weight and degree of deacetylation play pivotal role in skin tissue regeneration applications where a direct relation is established between these properties and rate of wound healing [27,28].

Curcumin, a natural drug with potent antioxidant and antibacterial attributes has been found to promote tissue regeneration by tissue remodeling, granulation, new tissue formation and collagen deposition [29]. Various carrier mediated drug delivery systems has been developed and tested for their potential in wound healing studies including polymer-curcumin nanoparticles [30,31], polymer-curcumin nanoemulsion gel [32], gel [33], self-assemble nanogel [34] and hydrogels [35,36]. Various attempts have also been made to develop such scaffolds in the form of sponges, polymeric films and nanofibers [37,38,39]. The curcumin has also been tested as a composite formulation for the purpose of accelerated wound healing applications [18,40]. Curcumin being photosensitive with poor bioavailability necessitates development of a suitable carrier enabling efficient penetration into skin with good bioavailability. 

Various chemical, physical, and biological methods are employed for the purpose of reducing the molecular weight of chitosan. Though, chemical method is a low cost method but possess the demerits of difficult process control and high cost of associate steps [41]. Similarly, physical methods like X-rays, gamma radiations and UV radiations though can easily be scaled up but they presents challenges like low quality of resultant product, unaffected degree of deacetylation and additionally special equipment’s are also required [42,43], while the enzyme based methods have high production cost [44]. In contrast, microwave is a cheap, nondestructive technique where with efficiently modifying the molecular weight chitosan without damaging the main chitosan chain and significantly reducing the degree of deacetylation of chitosan [45]. The main purpose of this study was to reduce chitosan molecular weight using microwave technique followed by physicochemical analysis of modified chitosan and later its use in the fabrication of curcumin nanoparticles as low molecular weight chitosan with low viscosity and high degree of deacetylation is envisaged to improve curcumin delivery into skin to hasten skin tissue regeneration following 2nd degree burn wound.

## 2. Materials and Methods

### 2.1. Chemicals

Chitosan (high molecular weight (HMW), molecular weight: 310 kDa to 375 kDa, degree of deacetylation: ~75 %, Sigma Aldrich, St. Louis, MO, USA)) was used as a starting material with curcumin (~95% purity, Zhejiang metals and minerals Import and Export Corporation, Hangzhou, Zhejiang Province, China) as a model drug for nanoparticle formulation. Ethanol (≥95% Sigma Aldrich, USA), monobasic potassium phosphate (Sigma Aldrich, St. Louis, MO, USA), disodium hydrogen orthophosphate (Merck, Darmstadt, Germany), hydrochloric acid (Merck, Germany), tripolyphosphate (TPP, Sigma Aldrich, USA), acetic acid (Sigma Aldrich, St. Louis, MO, USA), sodium chloride (Sigma Aldrich, St. Louis, MO, USA) and sodium hydroxide (Sigma Aldrich, St. Louis, MO, USA) were used in nanoparticle fabrication as adjuvant chemicals. All chemicals were used without any further purification.

### 2.2. Chitosan Molecular Weight Modification

The molecular weight of chitosan was reduced using already reported method [45]. Briefly, one accurately weighed gram of chitosan was added into accurately weighed amount of 49 g of 2% (*v*/*v*) acetic acid solution under ambient conditions with continuous magnetic stirring until complete dissolution which was then followed by the addition of 2 mL of 0.9% (*w*/*w*) sodium chloride solution for the purpose of increasing solution conductivity in order to hasten rapid chain breakage in a shorter time [46]. The mixture was then placed on the turntable of a microwave oven (LG, Model Number MS2022D, Beijing, China) in an off-center position. It was then subjected to microwave treatment at an irradiation power of 800 W for 5 min and 9 min intervals. Following microwave treatment, the solution was properly covered and left to cool at room temperature. The pH of the solution was then adjusted to 7.5 using 2N sodium hydroxide solution and then the polymer was precipitated by adding 40 mL of ethanol (96%). The precipitate was filtered, washed several times with distilled water and incubated in a hot air oven at 40 °C ± 1 °C for 24 h or until completely dry. The dried chitosan was then conditioned in a desiccator under ambient conditions for 24 h, triturated to form powder and then subjected to various characterization tests listed below.

### 2.3. Specific Viscosity (SV)

The solution viscosity of the modified chitosan was determined using an already reported method [47]. Briefly, a 0.5% (*w*/*w*) solution of each chitosan sample was prepared in 2% (*v*/*v*) acetic acid and filled into a U-tube viscometer (Ostwalds Viscometer, Zhejiang, China). The solution was sucked to an already marked distance and left to travel under gravity till the lower mark under ambient conditions. The time for solutions to travel from upper mark to lower mark were recorded. The same procedure was used for 2% (*v*/*v*) acetic acid solution as a reference. The relative solution viscosity of each chitosan sample was calculated using following relation:

(1) $$SV=\frac{F\;-F0}{F0}$$

where *F* is flow time of test solution, *F*0 is flow time of 2% (*v*/*v*) acetic acid solution.

Experiments were conducted at least in triplicate, and the results averaged.

### 2.4. Degree of Deacetylation (DD)

The DD of chitosan was determined using the vibrational spectroscopic technique. The respective chitosan samples were spread on a diamond crystal for attenuated total reflectance-fourier transform infrared spectroscopy (ATR-FTIR, UATR TWO, Perkin Elmer, Bukinghamshire, UK) and clamped to ensure close contact and high sensitivity. Each sample was scanned at a resolution of 4 cm^−1^ over a wavenumber region of 450 to 4000 cm^−1^.The DD of chitosan was calculated using the following equation:

(2) $$DD=100-\left[\frac{A1655}{A3450}\times \frac{100}{1.33}\right]$$

where A denoted ATR-FTIR absorbance value at 1655 and 3450 cm^−1^ wavenumbers ascribing amide-I of *N*-acetyl moiety and hydroxyl/amine moieties of the chitosan. The factor 1.33 denoted the value of A1655/A3450 for a fully *N*-acetylated chitosan [48]. Experiments were carried out at least in triplicate for each batch of samples and the results averaged.

### 2.5. Molecular Weight Analysis of Chitosan

The molecular weight analysis of HMW and low molecular weight (LMW) chitosan was done using gel permeation chromatography (Waters 1515 Isocratic HPLC pump, Waters Corporation, Hertfordshire, UK) equipped with dual a wavelength absorbance detector (2487, Waters Corporation, Hertfordshire, UK) and an injector (Waters 717 Plus Autosampler, Waters Corporation, Hertfordshire, UK) by means of a refractive index detector (Waters 2414 refractive index detector) as previously reported [49]. A styragel HR 5 tetrahydrofuran (THF) column (5 µm, 10–5 Å, 7.8 mm × 300 mm, Waters, Hertfordshire, UK) was used with tetrahydrofuran (THF) as the mobile phase. Samples were prepared by dissolving 5 mg of the polymer in 5 mL of 0.001% acetic acid (*v*/*v*) and filtered through nylon membrane (5 μm, Mountain Safety Research (MSR), Nylon Syringe Filter, Seattle, WA, USA) and 50 μl of it was then injected into the system. The flow rate of the mobile phase and the column temperature were kept at 1 mL/min and 40 °C, respectively. Experiments were carried out at least in triplicate and the results averaged.

### 2.6. LMW Chitosan-Curcumin Nanoparticles Preparation

LMW chitosan-curcumin nanoparticles were prepared by4 ionic gelation method, previously described [50] with some modification. Briefly, curcumin was separately dissolved in ethanol (96%) in a concentration of 1% (*w*/*v*) and chitosan was dissolved in 2% (*v*/*v*) acetic acid solution at pH 5.0 separately at concentrations of 0.3 and 0.4% (*w*/*v*), respectively. Two nanoparticles formulations were prepared viz. in one beaker, 3 mL of 0.3% chitosan solution was taken and added to 0.3 mL of curcumin solution, while, in another beaker, 3 mL of chitosan 0.4% was taken and added to 0.4 mL curcumin stock solution. Nanoparticles were spontaneously formed by adding 3 mL of TPP solution (0.1% *w*/*v*, in deionized distilled water) into the above solutions under a constant magnetic stirring (MS MP8 Wise Stir, Wertheim, Germany) at 700 rpm for 1 h at room temperature. The resultant modified chitosan-curcumin nanoparticles were harvested by ultracentrifugation (11,200× *g*) using an Optima L-100 XP Ultracentrifuge (Beckman-Coulter, Brea, CA, USA) at 18 °C for 1 h. The resultant pelleted nanoparticles were re-suspended in deionized distilled water, freeze dried at −48 °C for 3 to 4 days or until completely dry (Freezone loor top freeze dryer, Labconco, Kansas City, MO, USA) and subjected to further characterization tests.

### 2.7. Size and Zeta Potential

The particle size and zeta potential of the LMW chitosan-curcumin nanoparticles were measured by dispersing the nanoparticles in aqueous ethanolic solution through brief sonication and measured by method of photon correlation spectroscopy using Malvern Zetasizer Nano ZS 90 (Malvern Instruments Ltd., Malvern, UK) at 25 °C in a quartz cell and zeta potential cell with a detect angle of 90°, respectively. Experiments were conducted in triplicate and the results averaged.

### 2.8. Entrapment Efficiency and Drug Content

The entrapment efficiency and drug content of LMW chitosan-curcumin nanoparticles were determined by employing an indirect method of determining the free drug and the difference in total drug added and free drug determined. Briefly, the freeze-dried nanoparticles were resuspended in deionized water and filled into ultracentrifuge tubes and subjected to ultracentrifugation at 11,200× *g* using an Optima L-100 XP Ultracentrifuge (Beckman-Coulter, Brea, CA, USA) at 18 °C for 1 h. The obtained pellets were separated and washed with methanol twice to remove any unentrapped curcumin. The methanol rinse and the supernatant were combined and analyzed by HPLC (Waters 1515 Isocratic HPLC pump, Waters Corporation, Hertfordshire, UK) equipped with dual wavelength absorbance detector (2487, Waters Corporation, Hertfordshire, UK) and an injector (Waters 717 Plus Autosampler, Waters Corporation). A reversed-phase column (ODS Hypersil, 250 × 4.6 mm, 5 μm, Thermo Scientific, Loughborough, UK) fitted with a refillable C18 guard column (Upchurch Scientific, Oak Harbor, WA, USA) was used as the stationary phase for estimation of unentrapped drug. The mobile phase comprised of acetonitrile and trifluoro acetic acid (TFA, 0.2% *v*/*v*) in a 1:1 ratio at a flow rate of 1.5 mL/min with a detection wavelength of 430 nm. The experiment was repeated three times and the results averaged. The percentage of entrapped curcumin was calculated using the following relation:

(3) $$Percent\;entrappment\;efficiency\;=\frac{Total\;Cur\;added-unloaded\;Cur}{Total\;Cur\;added}\;\times 100$$

The total drug content of the nanoparticles was calculated using following relation:

(4) $$Percent\;Drug\;content=\frac{Loaded\;Cur}{Total\;Cur\;added}\;\times 100$$

The experiment was repeated three times and the results averaged.

### 2.9. Morphology

The Morphology of LMW chitosan-curcumin nanoparticles was analyzed using transmission electron microscopy (Tecnai Spirit, FEI, Eindhoven, The Netherlands) operated at an acceleration voltage of 80 kV. A total of 10 µL of samples (concentration 1 mg/mL) was placed on a 400-mesh copper grid coated with carbon and stained with uranyl acetate (2% *w*/*v*). The imaging was then performed, and respective sections photographed.

### 2.10. In Vitro Drug Release

The in vitro drug release pattern of the curcumin from nanoparticles was studied on vertical glass Franz diffusion cells (PermeGear Inc., Hellertown, PN, USA). Briefly, the receiving compartment of the Franz diffusion cell internal volume (12 mL) was filled with bubble free phosphate buffered saline (PBS) pH 7.4 to mimic wound pH with a surface area 1.76 cm^2^ enabling maximal drug solubility in the solvent maintained at 37 °C ± 1.0 °C with continuous magnetic stirring on a magnetic stirrer at 400 rpm. The Tuffryn^®^ membrane (0.2 µm pore size, HT Tuffryn^®^ membrane, Sigma Aldrich, St. Louis, MO, USA) was used as a barrier between the receiving and donor compartments. An accurately weighed 120 mg of nanoparticle was placed on membrane and the experiment run for 24 h. The sample aliquots of 1 mL were withdrawn at regular time intervals of 0, 0.5, 1, 2, 4, 6, 8, 12, 24 and 48 h and analyzed by HPLC (as discussed earlier) for the extent of drug release. Solvent equal in volume to volume withdrawn was added at each sampling point to maintain sink conditions. The cumulative amount of curcumin released and hence which permeated through per surface area (cm^2^) of membrane was calculated and plotted against time (h). The experiment was run in triplicate and the results averaged.

### 2.11. Drug Release Kinetics

The mechanism of drug release from the films was assessed by fitting drug release data into the Korsmeyer–Peppas equation as expressed by:

Mt/M∞ = Kt*^n^* (5)

where Mt/M∞ is a fraction of drug released at time t, k is the release rate constant and *n* is the release exponent. The 0.45 ≤ *n* corresponds to a Fickian diffusion mechanism, 0.45 < *n* < 0.89 to non-Fickian transport, *n* = 0.89 to Case II (relaxational) transport, and *n* > 0.89 to super case II transport [51].

### 2.12. Vibrational Spectroscopic and Thermal Analysis of Nanoparticles

The characteristics peaks of all samples (HMW chitosan, LMW chitosan, curcumin and LMW chitosan-curcumin nanoparticles) were recorded by an ATR-FTIR spectrophotometer (UATR TWO, Perkin Elmer, Bukinghamshire, UK). Each sample was placed on to the surface of the diamond crystal and clamped to ensure close contact and high sensitivity. All the samples were scanned over a wavenumber range of 450 to 4000 cm^−1^ with an acquisition time of 2 min. Each sample was analyzed three times and the results averaged.

Thermal analysis was also employed as an additional tool to the assess changes induced in the polymer by microwave treatment and to evaluate effective entrapment of curcumin inside the core of nanoparticles. Briefly, the changes in the transition temperature and enthalpies of all samples (HMW chitosan, LMW chitosan, curcumin and LMW chitosan-curcumin nanoparticles) were recorded via differential scanning calorimetry (PerkinElmer Thermal Analysis, Boston, MA, USA). An accurately weighed 2 to 3 mg of each sample was sealed in standard aluminum pan and heated from 0 °C to 300 °C under a continuous flow of nitrogen gas at rate of 40 mL/min. The characteristic peak temperature and enthalpy of the system were recorded. Each sample was analyzed three times and the results averaged.

### 2.13. Antimicrobial Activity

The antibacterial activity of LMW chitosan-curcumin nanoparticles was assessed using two selected bacterial strains i.e., *Staphylococcus aureus* (American Type Culture Collection (ATCC) 6538™)) and *Pseudomonas aeruginosa* (ATCC 9027™) under aseptic conditions (class II biological safety cabinet, Clyde Apac, Minto, Australia) via a well diffusion test. Briefly, a sterile swab was dipped in bacterial inoculum suspension and streaked on the surface of Mueller–Hinton agar following which multiple wells (diameter 6 mm) were made in agar plate carefully to ensure similar depth and diameter. Nanoparticles containing different drug concentrations (1000, 500, 250 μg/mL, pre-suspended in sterile deionized water) as well as blank nanoparticles were applied into wells using Gentamicin (20 μg/mL) as a positive control, distilled water as a negative control and plates incubated at 37 °C for 24 h (Memmert, Schwabach, Germany). The following day, zones of inhibition were measured. The results are presented as the mean of three experiments.

The minimum inhibitory concentration (MIC) and minimum bactericidal concentration (MBC) were determined using a broth microdilution assay [52,53]. Briefly, both bacterial inoculums were prepared and adjusted to cell density of 1–2 × 10^8^ CFU/mL. A total of 100 µL of Mueller–Hinton broth was dispensed into each target well of a 96-well plate followed by application of various nanoparticles formulations pre-suspended in sterile deionized water containing different concentrations of curcumin (1000, 500, 250, 125, 62.5 and 31.2 μg/mL) in triplicate with Gentamicin (20μg/mL) used as a positive control, distilled water as a negative control and broth without bacteria used as an environment control. The contents of wells were thoroughly mixed with a micropipette and incubated at 37 °C for 24 h. Following incubation, all the wells were visually inspected for the appearance where clear wells signify that bacterial growth has been inhibited. The minimum concentration of curcumin at which the wells remained colorless will be the MIC of the applied formulation. Following day, the contents of wells were then streaked onto agar plates and incubated at 37 °C for 24 h and observed for any bacterial growth. The minimum curcumin concentration at which no growth appears will be the MBC.

### 2.14. Cell Viability

Human Dermal Fibroblast-Adult (HDFa) cells were purchased from the Tissue Engineering Centre (Kuala Lumpur, Malaysia) and were cultured using Dulbecco’s Modified Eagle Medium (DMEM) supplemented with 1% Penicillin/Streptomycin, 5% fetal bovine serum in a T-75 flask and incubated at 37 °C under a 5% CO_2_ purge till confluence. Following incubation, cells were counted and 1 × 10^4^ cells per well in a 96 well culture plate were seeded and incubated overnight to ensure cell adhesion. On the following day, nanoparticles (1000, 500, 250, 125, 62.5 and 31.2 μg/mL, premixed with 100 µL of media) were applied and plates were incubated again for 24 h. The next day, media containing formulations were decanted off aseptically and 10 µL of MTT reagent was added into each well and the plates incubated again for 4 h where the live cells converted the MTT reagent to needle like formazan crystals which transit to the cell surface through the process of exocytosis from the endosomal/lysosomal compartment. The formed formazan crystals were dissolved by adding 100 μL of DMSO into each well, shaking for 20 min at 300 rpm followed by analysis on a UV microplate reader (Infinite^®^ 200 Pro NanoQuant, Tecan, Männedorf, Switzerland) at wavelength ah of 570 nm. The cell viability percentage was calculated using the following equation [54]:

(6) $$Cell\;viability=\;\frac{A1}{A2}\;\times 100$$

where A1 = Absorbance of treated cells; A2 = Absorbance of control cells.

The results are presented as a mean of six experiments.

### 2.15. Cell Migration Analysis

HDFa cells at a density of 1 × 10^4^ cells/well were seeded into a 6-well plate and incubated for 72 h in a humidified incubator maintained at 37 °C with 5% CO_2_ (ESCO CCL-170B-8 Singapore). Small linear scratches were created along the confluent monolayer of cells by gently scraping the surface using a sterile pipette tip. Cells were gently rinsed with sterile phosphate buffer saline (PBS) to remove cellular debris. Each well was applied with 1000 µg/mL formulation and blank nanoparticles applied separately and incubated again. The photographs of plates were taken at intervals of time 0, 24, 48 and 72 h using a digital camera connected to the inverted microscope (CK30-F200 Olympus, Tokyo, Japan). Plates without any treatment were used as a control. The time required for cells to fill the scratch completely was recorded for each application. All images were analyzed for the area left unfilled by the cells in the scratch after different time intervals via Image J using the unfilled area of scratch in photographs taken after no time as a standard scale. The experiment was repeated six times and the results compared. The percentage of wound closure was calculated as follows:

(7) $$Wound\;closure\;\left(\%\right)=\;\frac{w\left(0\right)-w\left(t\right)}{w\left(0\right)}\;\times 100$$

where *w*(0) is wound area at 0 time and *w*(t) is wound area at a specific time.

### 2.16. Statistical Analysis

All values are expressed as a mean of three readings with respective standard deviation. Student’s t-test or analysis of variance (ANOVA) followed by post-hoc analysis was used with the level of significance set at *p* < 0.05.

## 3. Results

### 3.1. Physicochemical Analysis of Parent and Modified Chitosan

Under the influence of microwaves, the chitosan chain scission took place at the main chain and at the amide linkage between the amino functional group of the parent chain and acetyl moiety. The HMW chitosan (molecular weight = 30,665 ± 245 Da; degree of deacetylation = 81.29 ± 6.9%) became shorter with 9 min microwave treatment (15,223 ± 182 Da, Student’s t-test, *p* = 0.002) and a higher degree of deacetylation (91.41 ± 8.2; Students t-test: *p* = 0.06). The LMW chitosan also had significantly lower solution viscosity than the HMW chitosan (parent chitosan = 13.466 ± 2.690 and 9 min = 2.009 ± 0.0164; *p* < 0.05). Similarly, the particle size of the powdered polymer also significantly reduced (Student t-test, *p* < 0.05) where for HMW chitosan the size of 4123.7 ± 421.4 nm reduced to 667.7 ± 145.7 nm and surface charge significantly increased (Student t-test, *p* < 0.05) from 21.4 ± 2.3 mV to 67.4 ± 6.2 mV with microwave scission of HMW chitosan due to reduced acetyl content (Table 1).

### 3.2. Physicochemical Analysis of Modified Chitosan Curcumin Nanoparticles

The size, polydispersability index (PDI), zeta potential of HMW chitosan, the LMW chitosan variant, drug content and entrapment efficiency of LMW chitosan-curcumin nanoparticles results with varying drug concentrations are given in Table 1. Pearson correlation analysis revealed that the concentration of chitosan is negatively related to size of the nanoparticles showing a strong indirect relation between the two (R = 0.959) while, in case of zeta potential, an indirect but weak relationship between the chitosan concentration and surface charge of the nanoparticles (R = 0.700)was detected.

### 3.3. Morphology

**Figure 1:**
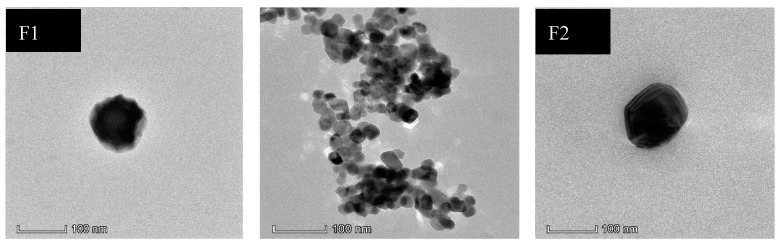
TEM pictographs of different LMW chitosan-curcumin nanoparticles.

The morphological analysis of the optimized LMW chitosan-curcumin nanoparticles results are given in Figure 1. The TEM analysis showed nanoparticles formed were spherical to square and pentagonal in shape with even geometry throughout their structure. The increase in curcumin quantity from 300 µg to 400 µg did not significantly alter the surface morphology where both the formulations showed smooth surface appearance. TEM results confirmed our data from dynamic light scattering where an increase in chitosan concentration did not significantly alter the nanoparticle structure and the overall morphology of square to rectangular and pentagonal remained independent of chitosan concentration [55].

### 3.4. In Vitro Drug Release

**Figure 2:**
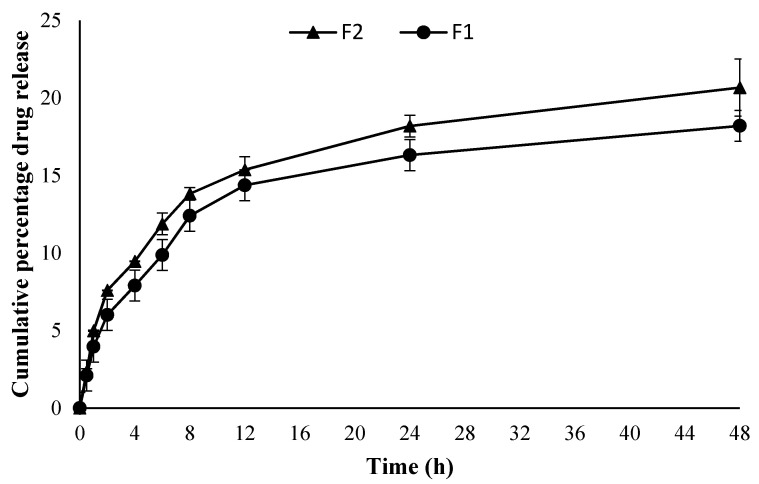
Cumulative percentage drug release (*n* = 3, ±SD).

The in vitro drug release behavior of the curcumin from both optimized formulations (F1 and F2) is given in Figure 2. Both the formulations showed a sustained drug release pattern with no significant difference between the two (ANOVA, *p* > 0.05) irrespective of the quantity of drug added into the formulation. The F2 sample containing a higher drug quantity showed a higher cumulative drug release pattern compared to the F1 formulation. The release was consistent from the very beginning of the release experiment for both formulations where F2 showed significantly higher cumulative drug released at the end of experiment i.e., 20.67% ± 1.84 µg/cm^2^·h compared to F1 where the same was found to be 18.12% ± 0.40 µg/cm^2^·h (Student’s t-test, *p* < 0.05).

### 3.5. Vibrational Spectroscopic Analysis

**Figure 3:**
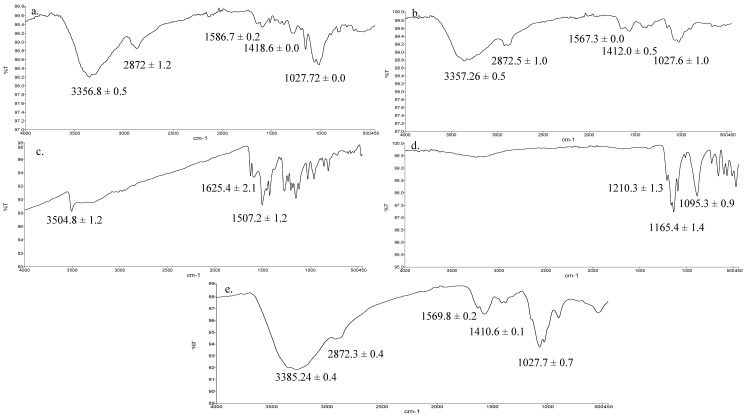
ATR-FTIR spectra of (**a**) high molecular weight (HMW) chitosan, (**b**) LMW chitosan, (**c**) pure curcumin, (**d**) tripolyphosphate (TPP), (**e**) formulation (F2) nanoparticles.

The ATR-FTIR spectra of HMW chitosan, LMW chitosan, TPP, pure curcumin and F2 nanoparticles are given in Figure 3. The principle peaks of chitosan were observed at 3358–3355 cm^−1^ which is mainly due to OH/NH stretching vibration, weaker bands between 2873–2871 cm^−1^ and 1587–1560 cm^−1^ corresponding to CH stretching and NH_2_ groups, respectively, and at 1027 cm^−1^ which arises due to C–O–C stretching (Figure 3a–c) [49]. The curcumin principle peaks were observed at 3505–3504 cm^−1^ and 1625 cm^−1^ and 1507 cm^−1^ which corresponds to OH stretching and aromatic (C=C) stretching (Figure 3d) [56]. The spectrum of TPP demonstrated principle signature peaks at 1210.3, 1165.4 cm^−1^ and 1095.3 cm^−1^ which demonstrate phosphate stretching (P–O–C) and phosphate vibration (P=O) and symmetric and asymmetric stretching vibration of the PO_3_ groups (Figure 3e) [57].

### 3.6. Thermal Analysis

**Figure 4:**
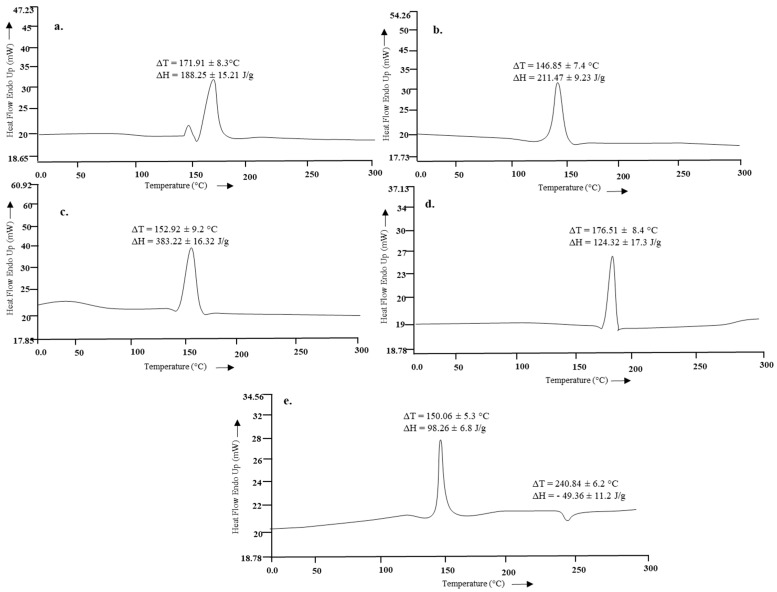
Thermal analysis of (**a**) parent chitosan, (**b**) microwave treated chitosan, (**c**) blank F2, (**d**) curcumin, (**e**) F2.

The thermal analysis results of HMW chitosan, LMW chitosan and the formulation are given in Figure 4. The endothermic peak of parent chitosan moiety at 171.91 °C ± 8.3 °C significantly reduced to 146.85 °C ± 7.4 °C depicting that microwave treatment could successfully scissor the chitosan chain into a small molecular weight moiety, but interestingly, the enthalpy of the system, instead of being reduced, significantly increased with microwave treatment (Student’s t-test, *p* < 0.05). 

### 3.7. Antimicrobial Analysis

**Figure 5:**
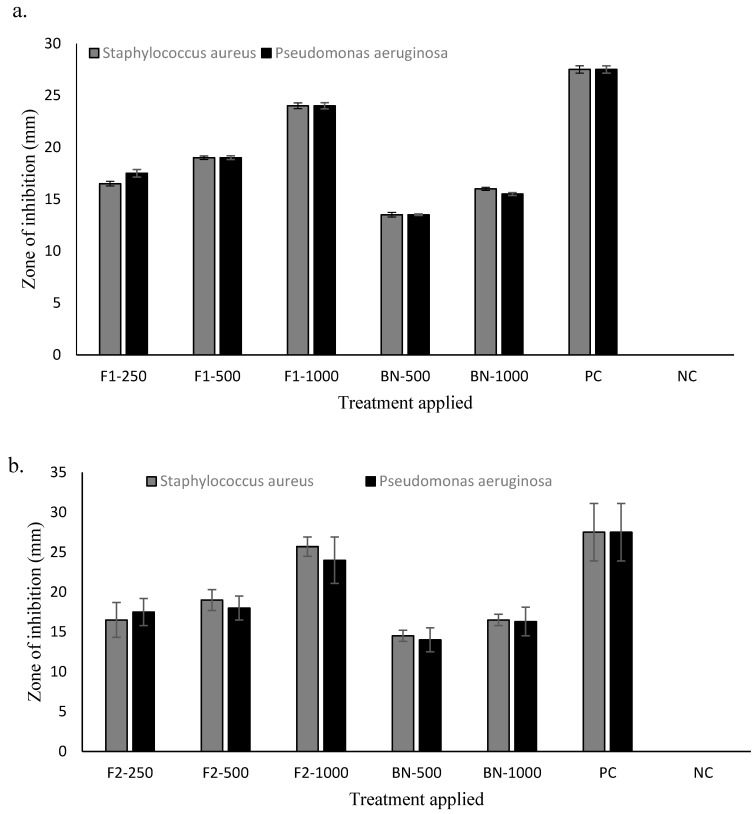
Antimicrobial activities of (**a**) F1 and (**b**) F2 different treatments applied: 250 µg, 500 µg, 1000 µg, BN = Blank nanoparticles, PC = positive control (Gentamicin), NC = negative control (distilled water).

The antimicrobial activities of drug loaded, and blank nanoparticles are given in Figure 2. Both the drug loaded formulations (F1 and F2) showed dose dependent antimicrobial activity against selected bacterial strains with significant effect (Student’s t-test, *p* < 0.05) observed for the F2 formulation containing higher quantity of chitosan used in nanoparticle fabrication. The formulated chitosan and curcumin nanoparticles were found to synergistically exert antimicrobial activity (Figure 5) where both the F1 and F2 formulations showed significant (Student’s t-test, *p* < 0.05) antimicrobial activity against *Staphylococcus aureus* and *Pseudomonas aeruginosa* compared to blank nanoparticles and comparable to the positive control. The MIC for drug loaded nanoparticles was found to be 250 µg for both formulations irrespective of chitosan quantity used while the MBC was found to be 1000 µg for F1 and 500 µg for F2 formulation demonstrating that, since F2 contained a higher concentration of chitosan, chitosan-curcumin exerted more synergistic bactericidal activity compared to F1.

### 3.8. Cell Viability and Cell Migration Analysis

**Figure 6:**
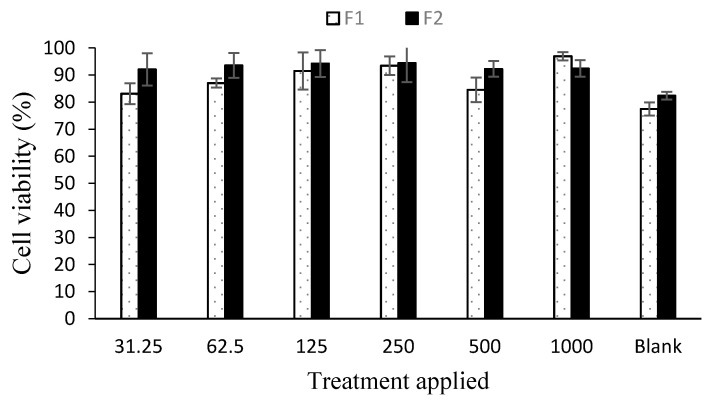
Cell viability analysis results (*n* = 6, ± SD).

**Figure 7:**
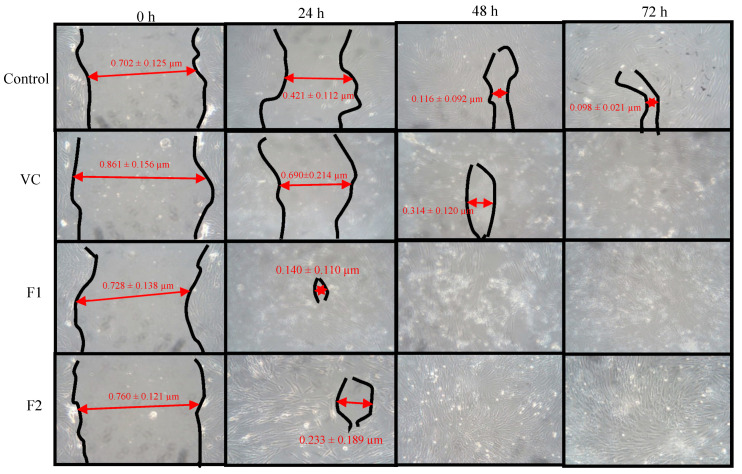
Cell migration analysis.

The drug loaded nanoparticles’ effect was evaluated using an adult human dermal fibroblast cell line using vehicle as a control. Both the formulations did not significantly affect the cell growth as the cell viability remained above 80% after 24 h post application due to low cytotoxicity ascribed to modified physicochemical attributes of chitosan (Figure 6). To assess the ability of chitosan and curcumin to promote cell proliferation, a cell migration test was carried out using human dermal fibroblasts with 1000 µg nanoparticle application up to 72 h using drug loaded as well as blank nanoparticles (Figure 7). The scratch area for F2 achieved full confluence within 48 h while F1 and the control within full confluence 72 h. The blank exhibited the slowest cell migration as the scratch area did not achieved full confluence within 72 h. The percentage wound closure was found to be 69.39 % (Student’s t-test, *p* < 0.05) post 24 h incubation compared to control and/or blank nanoparticles treatment where it was found to be 40.02% and 19.86%, respectively.

## 4. Discussion

Under the influence of microwaves, chitosan chain scission took place at the main chain and at the amide linkage between the amino functional group of the parent chain and acetyl moiety which significantly reduced the particle size and increased the surface charge with microwave scission of HMW chitosan due to reduced acetyl content. The molecular weight of the polymer is directly related to the size of the formulated nanoparticles where higher the molecular weight, the larger the nanoparticles achieved and vice versa. The skin drug and/or nanoparticle penetration is size dependent. Low molecular weight, low viscosity, smaller particle size and higher surface charge chitosan is envisaged to help produce smaller sized nanoparticles with efficient penetration into the skin and/or burn site and hence deliver the drug more effectively. Similarly, increase in the degree of deacetylation leads to formation of more free NH_2_ functional groups on the chitosan chain with reduced acetyl content, which is envisaged to impart more positive surface charge onto the chitosan chain due to protonation of NH_2_ by the free protons available in the acidic media, leading to a higher magnitude of the positive surface charge which is deemed favorable for the stability of nanoparticles due to mutual repulsion and hence prevents aggregation. Furthermore, skin is inherently negatively charged due to polar lipids, predominantly phospholipids, in the skin structure [58]. The negative charge on skin is deemed favorable to attract positively charged chitosan polymer moieties enabling adhesion and/or drug/nanoparticle penetration into the skin. Though nanoparticle size is inversely related to the chitosan concentration used in their fabrication, a situation where more free ${\mathrm{NH}}_{3}^{+}$ groups are available to react with TPP and having a chitosan concentration beyond 0.3% *w*/*v* are reported to increase the nanoparticle size [49,59,60]. Our findings were contradictory to this, where with the use of 0.4% *w*/*v* chitosan concentration, a slight decrease in the nanoparticle size (Student’s t-test, *p* = 0.260) was observed which can be attributed to the chitosan of low molecular weight used in the nanoparticle fabrication [61] (Table 1). Similarly, drug loading is also expected to further increase nanoparticles size, being an additional formulation moiety which is either adsorbed at the surface or entrapped in the nanoparticle core, and hence the particle size of the drug loaded nanoparticles was found to be significantly increased (Student’s t-test, *p* = 0.015) compared to blank nanoparticles [49,62,63] as shown in Table 1. Pearson correlation analysis revealed that the concentration of chitosan is negatively related to size of the nanoparticles, showing a strong indirect relation between the two (R = 0.959). The zeta potential analysis showed all nanoparticles bear high positive surface charge irrespective of the drug concentration added but significantly increase with increasing concentrations of chitosan (Student’s t-test, *p* < 0.05). The Pearson correlation analysis revealed an indirect but week relationship between the chitosan concentration and surface charge of the nanoparticles (R = 0.700). High surface positive charge not only ensures the stability of nanoparticles due to mutual repulsion but also guarantees enhanced antimicrobial activity through interaction with the bacterial cell membrane which is negatively charged. Moreover, blank and curcumin nanoparticles with high concentrations of chitosan (0.3% to 0.4% *w*/*v*) showed PDI values in the range of 0.5 to 0.6, which indicated narrow particle size distribution. The drug content of both formulations was found to be in the range of 99% to 100%, irrespective of the quantity of chitosan used in fabrication of nanoparticles which reflected no damage or spillage during experimentation with excellent entrapment efficiency of higher than 99% in both formulations.

Both the formulations (F1 and F2) showed a sustained drug release pattern (Figure 2). Curcumin is a poorly water-soluble and photosensitive drug. Encapsulation of curcumin in chitosan nanoparticles is envisaged to not only improve its higher cellular uptake but also improve stability and protection from photodegradation [64,65]. Sustained release of the drug with comparative good solubility in water (i.e., wound bed) is envisaged to promote skin regeneration with low frequency of dressing changing. The drug release data of F1 and F2 were fitted into the Korsmeyer–Peppas equation where both formulations showed a Fickian diffusion mechanism of drug release (F1 *n* = 0.350, R^2^ = 0.9514 and for F2 *n* = 0.336, R^2^ = 0.9634).

The vibrational analysis (Figure 3) indicated that microwave induced scission of chitosan does not significantly alter the main chain structure, confirming the nondestructive effect of microwave on parent chitosan while the nanoparticle spectra was missing the signature peaks observed for curcumin (i.e., aromatic stretching C=C) which demonstrated that the curcumin was entrapped in the core of nanoparticles instead of being adsorbed on the surface. The thermal analysis results (Figure 4) indicated that the glass transition temperature of chitosan and its oligomers are greatly affected by the number of amine groups available as they contribute towards hydrogen bonding between the chitosan chain and water [66]. Reduction of molecular weight leads to reduction of acetyl content and hence more NH_2_ functional groups available for hydrogen bonding between the chitosan chains and/or with the water; hence, this led to a significant increase in the energy required to induce a state transition (ΔH = 211.47 ± 9.23 J/g, Figure 4b). Thermal analysis of blank nanoparticles (Figure 4c) revealed a slight increase in the transition temperature and a significant increase in the enthalpy values, which is attributed to the formation of a cross linked structure by the use of tripolyphosphate [67]. Incorporation of curcumin into LMW chitosan nanoparticles insignificantly reduced the transition temperature of the chitosan moiety (Figure 4e), suggesting that that the drug is located between the polymeric chains, and increased the free volume and mobility of the system [68]. The transition temperature corresponding to curcumin moiety significantly increased from 176.51 °C ± 8.4 °C to 240.84 °C ± 6.2 °C (Figure 4d). Increase in the transition melting temperature of curcumin suggests an effective cross-linked polymer network was formed with curcumin entrapped inside the nanoparticle core and shift of curcumin melting temperature is envisaged due to the formation of additional interactive forces between the chitosan and curcumin moieties. Curcumin binds to the new polymeric network via hydrogen bonds with chitosan surface OH and/or NH_2_ functional groups [69] and/or electrostatic interaction with the former being the prominent binding force between chitosan and curcumin [70], thus promoting greater drug dispersion, stability and it may play pivotal role in increased drug loading into the nanoparticles.

The degree of deacetylation and molecular weight of the chitosan moiety are important parameters where a higher degree of deacetylation and low molecular weight are deemed favorable to interact with bacterial cell membrane and enable easy penetration into the bacterial cell, thus inserting a bactericidal activity. The antibacterial activity of chitosan is envisaged to be due to presence of charged groups in the polymer backbone which ionically interact with the bacterial membrane constituents resulting in hydrolysis of peptidoglycan, inciting leakage of intracellular electrolytes [71]. Burn wounds often become complicated by secondary bacterial infection where 61% mortality has been reported to be associated with *Staphylococcus aureus*, *Pseudomonas aeruginosa*, *Klebsiella sp.* and *Escherichia coli* based bacterial infections [72,73]. Curcumin is reported to possess significant antimicrobial activity against a wide range of Gram-positive and Gram-negative bacteria [74] while the chitosan moiety is also reported to possess antimicrobial activity against various bacterial strains [75]. The formulated chitosan and curcumin nanoparticles synergistically exerted antimicrobial activity (Figure 5) where both the F1 and F2 formulations showed significant (Student’s t-test, *p* < 0.05) antimicrobial activity against *Staphylococcus aureus* and *Pseudomonas aeruginosa* compared to blank nanoparticles and comparable to the positive control. The synergistic antimicrobial activity of chitosan and curcumin is envisaged to prevent infiltration of bacteria into the wound and hence prevent it from becoming complicated which will ultimately translate into better patient compliance and lower treatment cost.

The LMW chitosan-curcumin nanoparticles did not exert any significant cytotoxic effect, which is thought to be due to high degree of deacetylation of chitosan where it exhibits a stimulating effect on cells proliferation compared to counterparts with low degrees of deacetylation [76]. The degree of deacetylation of modified chitosan was significantly higher than parent chitosan (91.41 ± 8.2) which is hence deemed favorable in promoting skin tissue regeneration by facilitating dermal fibroblast proliferation. Curcumin exhibited cytotoxic effects in a dose dependent manner at concentrations higher than 5 µM [77]. Since our formulations contain curcumin in a significantly lower quantity compared to the reported concentration, hence no significant cytotoxic effect was observed in different nanoparticle applications. Both the tested formulations filled the scratch at significantly faster rates compared to the control and blank applications post 24 h incubation where the control and F1 formulations achieved full cell confluence after 72 h while F2 achieved full cell confluence within 24 h which can be attributed to the higher curcumin content embedded in core of the F2 formulation.

## 5. Conclusions

The molecular weight of chitosan was successfully reduced using microwave treatment. LMW chitosan generated small sized nanoparticles with positive surface charge and good dispersibility with the drug embedded inside the core of nanoparticles, thus facilitating sustained drug release in vitro through the mechanism of Fickian diffusion. The vibrational spectroscopic and thermal analysis results revealed that the chitosan main structure remained unaffected with microwave treatment but physical properties like transition temperature were significantly reduced. LMW chitosan-curcumin nanoparticles (F2) exerted synergistic antibacterial activity, with no significant cytotoxicity and enabled significantly faster scratch filling as well as cell confluence within 24 h post application comparable to control and/or blank treatment. LMW chitosan-curcumin nanoparticles with added synergistic antimicrobial activity and negligible cytotoxicity may prove beneficial in efficient delivery of curcumin into skin to hasten skin tissue regeneration following burn wounds.

## Figures and Tables

**Table 1:** Physicochemical analysis of polymer samples and low molecular weight (LMW) chitosan nanoparticles.

Formulation	Chitosan (%)	Curcumin (µg)	Size (nm)	Zeta Potential (mV)	PDI	Drug Content (%)	Entrapment Efficiency (%)
Parent chitosan	-	-	4123.7 ± 421.4	21.4 ± 2.3	1.0 ± 0.0	-	-
LMW Chitosan	-	-	667.7 ± 145.7	67.4 ± 6.2	0.72 ± 0.1	-	-
F1 blank	0.3	-	170.7 ± 2.9	46.4 ± 2.6	0.27 ± 0.05	-	-
F2 blank	0.4	-	223.7 ± 9.3	46.7 ± 1.6	0.5 ± 0.01	-	-
F1	0.3	300	279.7 ± 20.3	52.4 ± 1.50	0.67 ± 0.05	99.99 ± 1.39	99.93 ± 3.43
F2	0.4	400	259.2 ± 19.4	42.7 ± 1.53	0.54 ± 0.05	99.99 ± 0.34	99.96 ± 2.12
